# Investigating the impact of online language exchanges on second language speaking and willingness to communicate of Chinese EFL learners: a mixed methods study

**DOI:** 10.3389/fpsyg.2023.1177922

**Published:** 2023-05-12

**Authors:** Anju Zhou

**Affiliations:** School of Foreign Languages, Zhengzhou University of Technology, Zhengzhou, Henan, China

**Keywords:** online language exchanges, e-tandem, EFL context, speaking skills, WTC

## Abstract

**Introduction:**

The current study aims to investigate the impact of online language exchanges on the speaking skills and willingness to communicate (WTC) of Chinese postgraduate students in an advanced English program. The study compares two intact classes: e-tandem, who communicate with foreign English speakers through the Tandem language exchange application, and conventional, who participate in collaborative speaking tasks inside the class. The study also examines the attitudes and perceptions of the EFL learners toward the online language exchanges.

**Methods:**

58 Chinese postgraduate students were recruited from a second-year advanced English program and divided into two intact classes: e-tandem and conventional. The e-tandem group used the Tandem language exchange application to communicate with foreign English speakers online, while the conventional group participated in collaborative speaking tasks inside the class. The data were collected through the IELTS speaking module, WTC scale, and semi-structured interviews. The data were analyzed using descriptive and inferential statistics.

**Results:**

Both groups showed improvement in their speaking skills and WTC. However, the e-tandem group outperformed the conventional group. The findings indicate that online language exchanges have a positive impact on the speaking skills and WTC of EFL learners. The EFL learners also had positive attitudes and perceptions toward the online language exchanges, although some expressed reservations.

**Discussion:**

The study concludes that online language exchanges can be an effective tool for improving the speaking skills and WTC of EFL learners. The study also suggests that collaborative speaking courses in EFL settings should consider incorporating online language exchanges. However, the study also highlights the need to address the concerns and reservations expressed by some EFL learners regarding online language exchanges. Overall, the study has important pedagogical implications for EFL settings, suggesting that online language exchanges can enhance speaking skills and WTC.

## 1. Introduction

Second language (L2) speaking is a complex skill which requires learners’ integration of various language components such as vocabulary, grammar, and pronunciation to communicate effectively. However, in traditional English as a foreign language (EFL) classrooms, opportunities for students to practice speaking are often limited (Hwang et al., 2016). Moreover, learners may lack motivation and confidence in using their L2, resulting in low willingness to communicate (WTC) levels (Tai and Chen, 2020). Online language exchanges might provide a promising avenue for learners to engage in authentic conversations with native speakers and other L2 learners (Hagley, 2016, 2020; Watkins, 2019; O'Dowd, 2021), which can enhance their speaking competencies and increase their WTC levels (Rahimi and Fathi, 2022).

Virtual exchanges have been studied and explored from a wide range of angles (O’Dowd, 2018; Walker, 2018; Baroni et al., 2019; Hauck, 2019; Resnik and Schallmoser, 2019; Dooly and Vinagre, 2021; Lenkaitis and Loranc, 2021; Wu and Miller, 2021; Yeh and Heng, 2022). According to academics, all programs that enable online interpersonal interactions between students from various countries to learn languages or other courses should be referred to as virtual exchanges (O’Dowd, 2018; Hilliker, 2020). E-tandem, as a type of virtual exchange, is the online exchange between two language learners with various native languages who exchange their own language knowledge and abilities with their partners (Belz, 2003; Dooly and Sadler, 2013). Through online communication with their partners, learners can improve their language learning skills, such as speaking and writing skills (Gilmore, 2009; Bakar et al., 2013; Sangeetha, 2016; Fathi and Rahimi, 2022; Rahimi and Fathi, 2022).

Online exchanges are believed to address the time constraints and administrative restrictions which are common in speaking classes (Watkins, 2019; Wulandari, 2019; Zhao et al., 2020; Kinasih and Olivia, 2022; Pratiwi et al., 2022). Through online exchanges, learners can mediate each other’s speaking abilities (Rahimi and Fathi, 2022). Due to more exposure to and use of English in English-speaking contexts, learners also have more opportunities to connect with and develop their classmates’ speaking abilities outside of the classroom (Wu et al., 2017; Wu and Miller, 2021). Because of this, learners in non-English-speaking nations can have more chances to participate in group speaking activities, which are meant to help students improve their speaking abilities (Liu et al., 2023).

The majority of prior research on tandem language learning focused on peer interaction between tandem partners in various settings (Bower and Kawaguchi, 2011). For example, it has been revealed that e-tandem language learning can enhance learners’ engagement in interactive language learning activities (Ware and Kessler, 2016) and increase learners’ academic achievement (Jaime et al., 2013). However, as there is a limited number of research in the EFL context focusing on the authenticity of tandem learning in formal educational situations, more research on tandem learning is needed. On the other hand, insufficient research studies have concentrated on the role of e-tandem language learning in developing EFL learners’ oral proficiency and engagement.

In the meantime, English language learning has become growingly important in China, both as a tool for communication in the global economy and as a means to enhance one’s career prospects. While many Chinese EFL learners have opportunities to study English in a traditional classroom setting, there is a growing interest in online language exchanges as a way to improve speaking skills and increase willingness to communicate in English. However, despite the potential benefits of online language exchanges, little research has been conducted on their efficacy among Chinese EFL learners. Therefore, the aim of the present study is to investigate the impact of online language exchanges on the speaking skills and willingness to communicate of Chinese EFL learners.

Additionally, given the unique characteristics of the Chinese EFL context, including cultural factors and educational practices, it is important to explore how these factors may impact the effectiveness of online language exchanges. Additionally, while previous research has examined the impact of language exchanges on language learning outcomes, little is known about how Chinese EFL learners perceive and experience these exchanges. As a result, the present study investigated the effect of e-tandem language learning on Chinese EFL learners’ speaking performance and willingness to communicate. Willingness to communicate is referred to as learners’ motivation to participate in communicative speaking activities (Kruk, 2019). The findings implied that EFL learners communicate with English language speakers from various nations to improve their language learning skills, such as their speaking performance. Pedagogical implications are proposed for EFL teachers to engage EFL learners in more interactive, engaging, and authentic communicative speaking activities with online language learners of English.

## 2. Literature review

### 2.1. Theoretical framework

The interactive speaking activities in both classrooms in the current study adhere to Vygotsky’s (1984) social constructivism of learning. Vygotsky (1984) contends that interactions between pupils and smart peers can help them gradually internalize and activate higher forms of consciousness on their own. In other words, “every function in [students’] cultural development appears twice: first, on the social level, and later, on the individual level; first, between [students] (inter-psychological), and then, inside [individual students] (intra-psychological)” (Vygotsky, 1986, p. 57). The gap between [students’] developmental level as defined by autonomous problem solving and the greater level of potential development as identified through problem solving… in partnership with more capable peers is at the heart of Vygotsky’s social constructivism which is referred to as learners’ zone of proximal development (ZPD) or potential level of functioning (Vygotsky, 1984; Poehner et al., 2015). Learners can reach their ZPD via the support and interaction with other learners.

Several scholars suggest that learners’ interactive activities with peers can extend beyond interactions between less and more capable learners to interactions between students in pair and group work activities. In such activities, students can play the roles of both more and less capable people to help one another complete tasks (Storch, 2002; Kim, 2008). This can enable students to interact, co-construct their language skills, and reach their ZPD by sharing their diverse language abilities and knowledge. The current study employs Vygotsky’s social constructivism to engage EFL students in interactive speaking exercises in both traditional and e-tandem classes. However, the way the two groups conduct their speaking exercises distinguishes the two groups.

### 2.2. E-tandem language learning

E-tandem is defined as students’ online communication with either students from different geographical areas or native speakers of the target language (Telles and Vassalo, 2006; Dooly and Sadler, 2013). It entails the use of online communication technologies by language learners who are geographically distant to foster the development of (1) linguistic proficiency in a foreign language and (2) intercultural competence. In other words, by engaging in such communication, students can offer and receive real input and output while also developing their language learning skills (Belz, 2003, p. 68).

A number of studies addressed the effect of e-tandem language learning on different factors in the English language context (Garcia and Appel, 2016; Pomino and Gil-Salom, 2016; Fondo and Erdocia, 2018). For instance, Resnik and Schallmoser (2019) investigated the effect of e-tandem on language students’ language learning achievement. German students from the United Kingdom and the United States were paired with English speakers from an Austrian institution. The study attempted to pinpoint the connections between e-tandem language learning and foreign language enjoyment using information from 19 in-depth interviews. The results showed that most interviewees believed that e-tandem language learning increased their enthusiasm for studying other languages. Additionally, a variety of explanations for why students felt that learning a language via e-tandem was enjoyable were revealed, including having real-world conversations in the target language with native speakers, viewing one another as cultural interpreters, and a different power dynamic from language classroom contexts that put students at ease. The interviewees claimed that these features in particular boosted their happiness and raised their interest in using and studying the language. The results show that e-tandem language learning can be a tool to increase foreign language learners’ perceptions of enjoyment, and they also show that the social and private aspects of foreign language enjoyment appear to be connected.

Garcia and Appel (2016) studied the effect of an e-tandem program on learners’ communication with native English speakers and use of the target language. The e-tandem program included various discussion contexts and goals for the students to achieve. Data was collected through observations of video-recorded conversations of two language learners. The study found that learner interaction was influenced by task types and proficiency levels. Similarly, Kobayashi (2021) investigated the impact of e-tandem on students’ anxiety and reluctance to speak English when conversing with foreigners. The study focused on three Chinese undergraduate students in the United States and two Japanese undergraduate students in Japan through questionnaires before and after eight e-tandem sessions. The study found that students’ anxiety and unwillingness to speak in English significantly decreased after participating in the e-tandem program.

Lewis and Qian (2021) explored the use of e-tandem learning by adult distance learners with minimal second language skills. United Kingdom and Chinese learner engagement and development were checked via analysis of email correspondence, transcripts of conversations and interviews, surveys, and learning diaries. The results revealed that there was a lot of evidence for both intercultural learning and the acquisition of a second language. The findings provided pedagogical recommendations for how e-tandem language learning should be designed, with a focus on how to support e-tandem exchanges where participants have limited second language proficiency.

### 2.3. E-tandem and speaking performance

Several researchers have also corroborated the effective role of e-tandem language learning programs on learners’ oral skills (Elia, 2006; Canals, 2020; Lewis and Qian, 2021; Huilca Centeno, 2022). For example, Canals (2020) examined the motivation, communication, and teamwork of EFL learners as they interacted with native speakers in an online language exchange setting to see how their speaking abilities were developing. The results demonstrated that online language interaction improved the learners’ oral communication skills, motivation, and cooperation with other peers during the communicative tasks. Further analysis of the data revealed that learners with lower competence levels were more significantly affected by virtual language exchange in terms of oral abilities. In addition, Ware and Kessler (2016) investigated how interactions between second language learners are affected by online language exchange. According to the results, the students’ frequent information-seeking queries helped them get involved in partner-based interactive activities. The findings also suggested that the teacher may enhance student participation in the secondary learning setting by offering cues, fostering a secure online environment, and monitoring, directing, and interacting with the students.

In the study of Kawaguchi (2016), an e-tandem second language learning activity was evaluated using synchronous text-based computer-mediated communication. This exercise involved Japanese second language students in Australia and English second language students in Japan collaborating on a predetermined topic over three lessons, exchanging texts with each other. The results showed that engaging in meaning discussions and receiving corrective feedback had very positive effects. Furthermore, compared to casual conversation, the pace of the negotiation turns was significantly higher, and most students demonstrated growth in morphological and/or grammatical aspects. Therefore, while the use of synchronous computer-mediated communication for e-tandem learning in a second language can be beneficial, the instructor should carefully evaluate the activity and monitor student progress to ensure that it promotes language learning.

In a recent study conducted by Rahimi and Fathi (2022), the impact of e-tandem on the speaking abilities and willingness to communicate of EFL students was examined. EFL students in the e-tandem class used the Tandem language exchange application to communicate with foreign English speakers online, while those in the traditional class engaged in group speaking exercises in class. Results indicated that both groups showed improvement in speaking abilities and willingness to communicate, with the e-tandem group performing better than the traditional group. Qualitative analysis also produced several themes and categories representing the positive perceptions of the e-tandem speaking activities among students.

## 3. Purpose of the study

The research study demonstrated that e-tandem helped learners improve both their speaking abilities (Canals, 2020; Rahimi and Fathi, 2022) and their overall language learning (Garcia and Appel, 2016; Resnik and Schallmoser, 2019). The effect of e-tandem on EFL students’ speaking abilities and willingness to communicate in EFL contexts, however, appears to have not been sufficiently studied up to this point. In order to get further insight into the related literature, the current study investigated the effect of e-tandem on the speaking ability and willingness to communicate of Chinese EFL students.

## 4. Methods

The present study utilized an explanatory sequential mixed methods research design (Ivankova et al., 2006) to investigate the impact of online language exchanges on the speaking skills and WTC of Chinese EFL learners. The researcher began with the collection and analysis of quantitative data through the administration of the IELTS speaking module and the WTC scale. Then, the qualitative data were gathered through semi-structured interviews to explain and elaborate on the quantitative results. The qualitative data provided valuable insights into the learners’ perceptions and attitudes toward online language exchanges, resulting in a more comprehensive and in-depth understanding of the impact of online language exchanges on EFL learners.

### 4.1. Participants

To conduct this study, the convenience sampling method (Rose et al., 2019) was utilized to recruit two intact classes of Chinese EFL learners from a second-tier comprehensive university in central China. The sample consisted of 58 Chinese postgraduate students in psychology who were enrolled in a second-year mandatory advanced English program. The entry-level English proficiency required to join this program was a College English Test 4 (CET-4) which is equivalent to an IELTS score of 6.0. The participants were randomly assigned to either an experimental group (e-tandem class) consisting of 30 learners or a control group (conventional class) consisting of 28 learners. The participants were non-English major learners with Mandarin Chinese as their first language, and their ages ranged from 22 to 32 years with a mean age of 25.6 years. The course aimed to improve the EFL learners’ speaking skills, and none of the participants had prior experience using e-tandem for language learning. An experienced English instructor who was competent in technology use taught both the experimental and control classes.

In addition, the e-tandem (i.e., experimental) participants in this study comprised native and non-native English speakers from diverse regions worldwide who had a common objective of enhancing their English-speaking competencies or exchanging knowledge and cultural understanding with their e-tandem peers. This meant that the Chinese EFL students in the e-tandem group had the opportunity to participate in collaborative speaking tasks with their e-tandem partners, which facilitated the enhancement of their speaking abilities and understanding of their partners’ first language and culture. Additionally, the Tandem application provided filters to select appropriate partners for collaborative speaking tasks according to the Chinese EFL learners’ interests, ensuring that their e-tandem partners shared interests.

### 4.2. Instruments

The researcher used the IELTS speaking skill test to evaluate the Chinese EFL learners’ speaking abilities. This test is comprised four equally weighted areas, namely fluency and coherence, vocabulary, grammatical range and accuracy, and pronunciation. The IELTS speaking test topics were used to evaluate the learners’ performance in each area. The IELTS Speaking Band Descriptors were used to assign a score from 1 to 9 to each learner in each area of speaking skill. These scores were added up and then divided by four to determine the global speaking score of each student. To ensure consistency, the learners’ speaking skills were assessed by two trained raters, including the researcher and another experienced EFL speaking instructor. The inter-rater reliability was acceptable at 0.83, as measured by the Cohen’s kappa coefficient.

The L2 WTC scale included eight items that were taken from Yashima (2009) and aimed to measure the participants’ L2 WTC in real-life communication situations. These items were designed to prompt the participants to think about their willingness to communicate in various scenarios, such as when you have a group discussion in an English class. The items were rated on a six-point Likert scale, with responses ranging from 1 (definitely not willing) to 6 (definitely willing), and the even-numbered scale was used to prevent neutral responses.

Finally, to investigate the participants’ perceptions and attitudes toward the overall process and its impact on their speaking skills, semi-structured interviews were carried out. After transcribing the interviews, the technique of member checking was used to validate the accuracy and credibility of the obtained results.

### 4.3. Data collection

For the pre-test, both the experimental and control groups of EFL learners were assessed using the Cambridge IELTS 9 speaking test. This test required the learners to speak about themselves and familiar topics for around 5 min, followed by a specific topic for which they were given 3 min to prepare notes. The learners were evaluated based on the IELTS Speaking Band Descriptors, which covered the four aspects of fluency and coherence, lexicon, grammatical range and accuracy, and pronunciation. After the pre-test, both groups were exposed to speaking instruction which was tailored to enhance IELTS speaking competencies. The experimental group engaged in e-tandem exchanges for out-of-class speaking practice, while the control group had out-of-class speaking practice in a language lab.

The Chinese EFL students in the experimental group were introduced to the Tandem application through instructional videos and were given 10 days to find suitable language partners. This period served as a trial to familiarize themselves with the e-tandem method and the application. During each session, learners started with warm-up activities by discussing topics introduced in the coursebook with the researcher/instructor before collaborating on speaking tasks in groups of four or five. After class, the students were involved in collaborative speaking tasks with their foreign peers using the Tandem application, focusing on the same topics as the previous session. The selection of e-tandem partners was based on similar interests and a willingness to follow the learners’ selected topics for group speaking tasks. These topics were already integrated into the Tandem application. The students used various communication modes, such as text messages, audio or video calls, and voice notes. Finally, the students were requested to think over their experiences of online exchanges and share their perceptions.

Similarly, the control group received the identical course materials and speaking instruction content as the e-tandem group but did not engage in telecollaboration tasks with foreign and native English speakers. Instead, they discussed the speaking topics with their instructor and collaborated with their classmates during class time. After class, the conventional learners gave face-to-face presentations inside the classroom. To avoid exhaustion and accommodate time preferences, the presentation sessions were spread over several sessions per week, and participants were free to attend one or more sessions. Each presenter had a 15-min preparation time before their presentation, and their classmates provided feedback and comments on their performance.

### 4.4. Data analysis

In order to evaluate the effect of e-tandem and traditional speaking instruction on EFL learners’ speaking skills and compare the effectiveness of these two methods, one-way ANCOVA was employed. The ANCOVA analysis was also used to investigate the impact of and compare the two types of instruction in affecting the learners’ WTC. To control for any pre-existing differences between the two groups, learners’ pre-test marks were considered as covariates. Thematic analysis was used to analyze the transcribed interviews. Inter-rater reliability was ensured by the researcher and two other expert L2 researchers who checked the coding and labeling for accuracy and consistency. The disagreements among the researchers were discussed and resolved.

## 5. Results

### 5.1. Quantitative data

The first step in analyzing the data was to use descriptive statistics to compare the mean scores of the EFL learners’ speaking skills in both the experimental and control classes before and after the study. Table 1 displays the results of these descriptive statistics, which show some differences in the mean scores for fluency between the pre- and post-tests for both groups. Additional statistical analyses, such as paired samples *t*-tests and one-way ANCOVA, were conducted to determine if these differences were significant. The paired samples *t*-tests were used to compare the pre- and post-test mean scores for each group.

**Table 1:** Descriptive statistics.

	Group	*N*	Mean	Std. deviation
Fluency 1	Experimental	30	4.9933	0.56569
	Control	28	5.1250	0.60965
Fluency 2	Experimental	30	5.9200	1.04240
	Control	28	5.3304	0.83961
Vocabulary 1	Experimental	30	4.5550	0.62330
	Control	28	4.6471	0.60578
Vocabulary 2	Experimental	30	5.9517	0.98139
	Control	28	5.1007	0.69224
Accuracy 1	Experimental	30	5.5630	0.72776
	Control	28	5.3364	0.74961
Accuracy 2	Experimental	30	6.5567	0.83364
	Control	28	5.6786	0.78733
Pronunciation 1	Experimental	30	4.7020	1.21316
	Control	28	4.4389	0.65566
Pronunciation 2	Experimental	30	6.5433	0.87600
	Control	28	5.5768	0.77573
Global speaking 1	Experimental	30	4.1071	0.35068
	Control	28	4.0931	0.28632
Global speaking 2	Experimental	30	5.9428	0.59399
	Control	28	5.4804	0.48586
WTC 1	Experimental	30	3.0733	0.59766
	Control	28	2.8714	0.67651
WTC 2	Experimental	30	4.2567	1.14038
	Control	28	3.5125	0.79810

The results displayed in Table 2 suggest that there were substantial changes in the EFL learners’ speaking skills between their pre- and post-test scores in the experimental group, indicating that e-tandem had a significant impact on enhancing their speaking abilities. For example, the difference between the pre-test mean score of fluency and the post-test mean score of fluency was −0.92, which represents a significant improvement in the EFL learners’ fluency, as evidenced by the value of *p* [t (29) = −4.82, *p* = 0.00]. Paired samples *t*-tests were also used to determine the distinctions between the pre- and post-test mean scores of the control group learners. As seen in Table 2, the outcomes of paired samples *t*-tests indicates that the traditional instruction was useful in boosting the EFL learners’ speaking skills. Then, to compare the two groups’ improvements in speaking skills, ANCOVA was conducted. Table 3 shows the results of ANCOVA that investigates the differences between e-tandem and control classes in boosting the EFL learners’ fluency.

**Table 2:** Paired samples *t*-test results.

Group					*t*	Df	*Sig.*
			Mean	*SD*			
Experimental	Pair 1	Fluency	−0.92667	1.05238	−4.823	29	0.000
	Pair 2	Vocabulary	−1.39667	0.83166	−9.198	29	0.000
	Pair 3	Accuracy	−0.99367	0.52893	−10.290	29	0.000
	Pair 4	Pronunciation	−1.84133	1.69624	−5.946	29	0.000
	Pair 5	WTC	−1.18333	1.02051	−6.351	29	0.000
	Pair 6	Global Speaking	−1.83571	0.44085	−22.807	29	0.000
Control	Pair 1	Fluency	−0.20536	0.44369	−2.449	27	0.021
	Pair 2	Vocabulary	−0.45357	0.32978	−7.278	27	0.000
	Pair 3	Accuracy	−0.34214	0.27087	−6.684	27	0.000
	Pair 4	Pronunciation	−1.13786	1.05675	−5.698	27	0.000
	Pair 5	WTC	−0.64107	0.58989	−5.751	27	0.000
	Pair 6	Global Speaking	−1.38730	0.30824	−23.815	27	0.000

**Table 3:** ANCOVA results of fluency.

Source	Type III sum of squares	Df	Mean square	F	Sig.	Partial eta squared
Corrected model	18.646	2	9.323	13.883	0.000	0.335
Intercept	1.415	1	1.415	2.108	0.152	0.037
Fluency 1	13.610	1	13.610	20.267	0.000	0.269
Group	7.009	1	7.009	10.437	0.002	0.159
Error	36.935	55	0.672			
Total	1897.493	58				
Corrected total	55.580	57				

The findings presented in Table 3 suggest that there were significant disparities in the post-test scores of fluency skill between the experimental and control groups. This confirms that the experimental condition was more efficient in enhancing the EFL learners’ fluency skills than the traditional instruction. Additionally, Table 4 displays the outcomes of the ANCOVA conducted to compare the two groups’ effectiveness in enhancing vocabulary skills. It indicates that the e-tandem significantly improved the EFL learners’ vocabulary skills compared to the conventional group, as significant differences were observed in the post-test marks of vocabulary skills, even after controlling for pre-test scores. Likewise, Table 5 demonstrates the results of the ANCOVA, which was conducted to examine the disparities between the two groups in improving accuracy skills.

**Table 4:** ANCOVA results of vocabulary.

Source	Type III sum of squares	Df	Mean square	F	Sig.	Partial eta squared
Corrected model	28.491	2	14.245	34.265	0.000	0.555
Intercept	1.620	1	1.620	3.897	0.053	0.066
Vocabulary 1	18.003	1	18.003	43.304	0.000	0.441
Group	12.613	1	12.613	30.338	0.000	0.356
Error	22.866	55	0.416			
Total	1832.023	58				
Corrected total	51.356	57				

**Table 5:** ANCOVA results of accuracy.

Source	Type III sum of squares	Df	Mean square	F	Sig.	Partial eta squared
Corrected model	38.078	2	19.039	104.922	0.000	0.792
Intercept	1.013	1	1.013	5.580	0.022	0.092
Accuracy 1	26.911	1	26.911	148.303	0.000	0.729
Group	6.260	1	6.260	34.496	0.000	0.385
Error	9.980	55	0.181			
Total	2229.480	58				
Corrected total	48.058	57				

Table 5 shows that significant differences were observed between the experimental and control classes in terms of accuracy skill improvement. This indicates that the experimental treatment was more useful in fostering the EFL learners’ accuracy than the traditional instruction.

Table 6 illustrates the ANCOVA results, which examined the differences between the experimental and control classes’ pronunciation skill. The findings indicate that the treatment was substantially effective in enhancing the EFL learners’ pronunciation skills.

**Table 6:** ANCOVA results of pronunciation.

Source	Type III sum of squares	Df	Mean square	F	Sig.	Partial eta squared
Corrected model	15.523	2	7.761	11.693	0.000	0.298
Intercept	119.649	1	119.649	180.252	0.000	0.766
Pronunciation 1	1.993	1	1.993	3.002	0.089	0.052
Group	14.706	1	14.706	22.155	0.000	0.287
Error	36.508	55	0.664			
Total	2193.773	58				
Corrected total	52.031	57				

Table 7 further confirms the superiority of the experimental class over its traditional counterpart in improving the EFL learners’ global speaking performance, as significant differences were observed between the two groups’ post-test scores of total speaking performance after controlling for the covariates.

**Table 7:** ANCOVA results of global speaking.

Source	Type III sum of squares	Df	Mean square	F	Sig.	Partial eta squared
Corrected model	11.799	2	5.899	41.052	0.000	0.599
Intercept	0.158	1	0.158	1.102	0.298	0.020
Global1	8.702	1	8.702	60.556	0.000	0.524
Group	2.870	1	2.870	19.969	0.000	0.266
Error	7.904	55	0.144			
Total	1917.112	58				
Corrected total	19.702	57				

The researchers conducted quantitative statistical analyses to investigate the effect of experimental and traditional instructions on EFL learners’ WTC. Descriptive statistics was utilized to examine the pre- and post-test mean scores of WTC scale in both groups, and the results are presented in Table 1. The table shows differences between the pre- and post-test mean scores of WTC in each group, as well as differences between the two groups’ post-test mean scores of WTC. The researchers then performed paired samples *t*-tests to determine whether significant differences existed between the pre- and post-tests in each group. Table 2 presents the results of paired samples *t*-tests for the experimental group, indicating substantial changes in pre- and post-test marks of WTC, which suggest that the experimental program was effective in enhancing the learners’ WTC. The researchers also conducted paired samples *t*-tests for the conventional group (see Table 2).

The results demonstrate that significant differences existed between the pre- and post-test scores of WTC among the control group learners, indicating that the traditional speaking instruction also played a vital role in improving the EFL learners’ WTC. Nevertheless, to compare the two groups’ WTC improvements, ANCOVA was conducted. The results of this analysis are presented in Table 8, which examines the differences between the experimental and control groups in improving the EFL learners’ WTC. Table 8 shows that after controlling for the pre-tests as covariates, significant differences were found between the two groups’ post-test marks of WTC, demonstrating that the experimental instruction was more useful than the conventional instruction in enhancing the EFL learners’ WTC.

**Table 8:** ANCOVA results of WTC.

Source	Type III sum of squares	Df	Mean square	F	Sig.	Partial eta squared
Corrected model	23.938	2	11.969	16.882	0.000	0.380
Intercept	4.801	1	4.801	6.771	0.012	0.110
WTC 1	15.918	1	15.918	22.451	0.000	0.290
Group	4.670	1	4.670	6.586	0.013	0.107
Error	38.994	55	0.709			
Total	943.942	58				
Corrected total	62.932	57				

### 5.2. Qualitative data

In the qualitative phase of this research, the purpose was to explore the perceptions of participants of the experimental group toward e-tandem. Thematic analysis was used to identify recurring patterns and themes in the data. The following themes were identified:

#### 5.2.1. Comfort and confidence

Participants spoke about their initial hesitancy and lack of confidence in using video chat to practice their speaking skills. However, over time, they began to feel more comfortable and confident in their abilities. For example, one participant said, “At first, I was hesitant to use video chat because I wasn’t confident in my speaking skills. However, after some time, I started feeling more comfortable and it became easier for me to have a conversation with my partner.”

#### 5.2.2. Correction and technical difficulties

The experimental group students appreciated the correction function on the app as it helped them understand their mistakes and learn from them. Nevertheless, some technical difficulties with the photo uploading option were reported. For example, one participant said, “I appreciated the correction function on the app because it helped me understand my mistakes and learn from them. However, there were some technical difficulties with the photo uploading option.”

#### 5.2.3. Finding suitable partners

Some students reported that finding a suitable tandem partner who was dedicated to learning the language was challenging. Some people seemed to be more interested in flirting than actually practicing the language. For example, one participant said, “It was challenging to find a suitable tandem partner who was dedicated to learning the language. Some people seemed to be more interested in flirting than actually practicing the language.”

#### 5.2.4. Colloquial expressions and motivation

EFL students found it difficult to understand some of the colloquial expressions used by their partners at first. However, this motivated them to improve their listening and speaking skills in order to communicate better with their partners. For example, one participant said, “I found it difficult to understand some of the colloquial expressions used by my partners at first. However, this motivated me to improve my listening and speaking skills in order to communicate better with them.”

#### 5.2.5. Enjoyment and motivation

Also, students reported that they enjoyed talking with some partners more than others. This made them more motivated to practice their speaking and to prepare for their next conversation. For example, one participant said, “I enjoyed talking with some partners more than others. It made me more motivated to practice my speaking and to prepare for our next conversation.”

#### 5.2.6. Personal boundaries

Few students also reported that some people asked personal questions that they did not feel comfortable answering. This made them realize the importance of setting boundaries in language exchanges. For example, one participant said, “There were some people who asked personal questions that I did not feel comfortable answering. It made me realize the importance of setting boundaries in language exchanges.”

#### 5.2.7. Different dialects and accents

The participants reported that they noticed some of their partners from the Middle East used very formal language that was different from what they were used to hearing. It was interesting to learn about the different dialects and accents of English. For example, one participant said, “I noticed that some of my partners from the Middle East used very formal language that was different from what I was used to hearing. It was interesting to learn about the different dialects and accents of English.”

#### 5.2.8. Tandem as a supplement

Additionally, a number of participants reported that while they preferred more traditional classroom settings, Tandem was a useful supplement to their language learning. It allowed them to practice speaking and connect with people from around the world. For example, one participant said, “I prefer more traditional classroom settings, but I found Tandem to be a useful supplement to my language learning. It allowed me to practice speaking and connect with people from around the world.”

## 6. Discussion

Drawing on Vygotsky’s (1984) social constructivism of learning, the present study investigated the impact of using online language exchanges via e-tandem on the speaking performance and willingness to communicate of EFL students. The findings indicated that e-tandem class enhanced the speaking performance of EFL students and outperformed its conventional counterparts in that regard. The findings are in line with those of Canals (2020) and Rahimi and Fathi (2022) who confirmed the positive impact of e-tandem language learning programs on EFL students’ speaking performance. The findings might be due to the effect of the foreign speakers of English which, as a new experience, motivated the e-tandem learners to have more engaging communicative speaking activities with their online partners and enhance their speaking abilities accordingly. The online experience of the e-tandem class also engaged the learners of the e-tandem class to communicate with their partners whenever and wherever they thought was convenient for them, which could have a positive impact on their speaking performance.

Following Vygotsky’s (1984) social constructivism of learning, the learners’ speaking abilities in the e-tandem class were mediated by their online partners. Initially, the learners had communicative speaking activities with their online partners in an interactive manner which, according to Vygotsky (1984), could help them regulate their own speaking abilities and those of their partners. The learners’ other-regulation via the interactive speaking activities gradually contributed to the learners’ self-regulation of speaking abilities. The learners who achieved their self-regulation could act autonomously and perform their speaking tasks without the contribution of other learners. The findings drew on the fact that the e-tandem learners were more successful than the conventional learners in achieving their self-regulation of speaking performance.

In harmony with the findings of Liu et al. (2023), the aforementioned findings might be also due to the cultural elements the learners acquired during their communicative speaking interactions with their online foreign speakers of English. As the learners and their online partners had different cultural backgrounds, they could share their cultural knowledge with their partners which was thought to be engaging for the learners. The enthusiasm for learning new cultural elements could also contribute to the e-tandem learners’ speaking performance. The e-tandem learners’ successful internalization of cultural elements might be another reason behind the e-tandem learners’ more successful speaking performance.

The findings further revealed that the e-tandem program improved the learners’ willingness to communicate and outperformed its conventional counterpart. The findings are in agreement with the findings of Rahimi and Fathi (2022) who corroborated the beneficial effects of the e-tandem program in enhancing EFL learners’ willingness to communicate. E-tandem learners were more willing to communicate and utilize the target language in real-world situations and develop their speaking skills. E-tandem learners were also more open to participating in communicative speaking tasks and would communicate outside of the classroom with their online partners, which could significantly contribute to their speaking performance and willingness to communicate. Moreover, the e-tandem learners were willing to communicate and achieve their autonomy in speaking tasks, which could corroborate their more successful performance in linguistic skills and pursue their language learning goals.

The findings may be also the result of the students’ stronger drive to have interactive speaking activities via e-tandem language learning model and their perception that working in an e-tandem program was far more motivating than working alongside their classmates during class time. Rahimi and Fathi (2022), among other studies, indicated that these kinds of interactions affected motivation and WTC with native speakers. Therefore, interacting with native speakers seems to have the benefit of increasing learners’ motivation to communicate and work interactively (Garcia and Appel, 2016). When students perceive a genuine need for communication, they are much more likely to be willing to engage in it and view the activities assigned to them to be more interesting (Kobayashi, 2021). The dual positions that each student assumed—as both a learner and an expert—could also have a favorable impact on their speaking performance and willingness to communicate. That is, although the learners learned different English language items from their online partners, they taught their online partners about the rules and items of their own native language, which further motivated the learners to have more engaging interactive speaking activities with their online partners.

It is suggested that implementing an e-tandem program can engage students in a comfortable online space where they can interact with English speakers all over the world and increase their speaking ability and willingness to converse more successfully. According to the present study, EFL students actively participate in interactive speaking activities with their online partners, which improves their speaking abilities and willingness to communicate more properly. It is thought that these collaborative speaking activities help learners develop their ability to self-regulate their speaking and communication behaviors. According to Vygotsky (1986), learners with various aptitudes and talents can aid one another in reaching their optimal level of functioning. In the present study, the online course assisted the students in having more successful engaging communicative speaking tasks (other-regulation) by enhancing favorable impressions of their online members’ cultures and skills (Liu et al., 2023) for cooperative and comprehension reasons. This is in line with Rahimi and Fathi’s (2022) findings. Because the participants in the current study had a variety of cultural backgrounds, they were able to significantly improve their partners’ cultural self-regulation, and hence, their speaking performance and willingness to communicate.

The findings of the qualitative data analysis suggest that online language exchanges revealed the benefits and challenges of using e-tandem for language learning. One of the major benefits identified was the increase in confidence and comfort levels of the participants in speaking the target language. Participants reported that, although they were initially hesitant, the experience of using e-tandem helped them to overcome their fear of making mistakes and gain confidence in their language abilities. This finding is consistent with previous research that has shown the importance of creating a supportive environment for language learners to practice their skills without fear of judgment (Shih, 2010; Wang and Vásquez, 2012; Yousefifard and Fathi, 2021).

Another benefit highlighted was the opportunity to practice with native speakers, which allowed participants to improve their pronunciation and vocabulary in a more authentic context. The use of colloquial expressions by partners also presented a challenge for participants, but motivated them to improve their listening and speaking skills to better communicate with their partners. The present study’s identification of the opportunity to practice with native speakers aligns with previous research that has shown that interactions with native speakers via technology can provide learners with valuable opportunities to improve their language skills (Yeh and Lai, 2019; Mumford and Dikilitaş, 2020; Russell, 2020). Similarly, Marull and Kumar (2020) also reported that online language exchanges provided opportunities for learners to practice authentic language in real-life situations. However, the study also identified some challenges associated with the use of e-tandem for language learning. One such challenge was the difficulty in finding suitable tandem partners who were dedicated to learning the language. Some participants reported encountering partners who were more interested in flirting than language practice. This highlights the importance of setting clear boundaries and expectations for language exchanges. The identification of the importance of setting clear boundaries and expectations for language exchanges aligns with previous research that emphasizes the significance of establishing clear goals and expectations for language exchange programs to ensure their effectiveness (Sevy-Biloon and Chroman, 2019; Yeh and Heng, 2022).

Another challenge identified was the technical difficulties that some participants faced while using the app, such as problems with photo uploading. This suggests the need for continuous improvement of the technological features of e-tandem to enhance the user experience. This finding supports previous research that underscored the significance of ensuring the usability of language learning technologies to enhance the user experience (Fernández-López et al., 2013; Chen et al., 2021). Overall, the findings suggest that e-tandem can be a useful supplement to traditional classroom methods for language learning, providing learners with opportunities to practice speaking and connect with people from around the world. However, the challenges identified in this study emphasize the need for careful consideration of the design and implementation of e-tandem programs to ensure their effectiveness and usability for language learners.

## 7. Conclusions and implications

The current research aimed to investigate the impact of online language exchanges on the speaking skills and WTC of Chinese EFL learners. The researcher concentrated on an e-tandem language learning program which was designed to help EFL learners improve their speaking performance and willingness to communicate. Following Vygotsky’s social constructivism, the findings indicated that the e-tandem class was more effective in improving the EFL learners’ speaking performance and willingness to communicate than the conventional class.

The study’s findings showed that the e-tandem class had a beneficial impact on the students’ speaking abilities and willingness to communicative. The results may be directly related to the students’ interactions with online foreign English speakers, which they perceived as being more enthusiastic than in-person speaking learner interactions. The results suggested several instructional implications. It is advised to use the e-tandem class in EFL interactive speaking courses since it is consistent with contemporary notions of student-centeredness and produces positive results for EFL students’ speaking performance and willingness to communicate. According to the research, EFL educators should encourage EFL teachers and students to apply an e-tandem program for interactive speaking activities so that the EFL students’ speaking abilities and willingness to communicate can be enhanced.

It is suggested that EFL teachers create an e-tandem class to give EFL students interesting interactive speaking activities with online foreign English speakers. This allows EFL students to engage in more cooperative and interpretive communicative speaking tasks in order to enhance their speaking ability and their willingness to communicate more effectively. EFL students could have and get more peer speaking mediations on the speaking assignments by having an e-tandem classroom designed specifically for interactive speaking activities. The students can also make greater use of engaging communicative speaking tasks, improve their speaking performance, and increase their willingness to communicate as a result. Given the importance of oral communication in English, this finding is particularly relevant in the Chinese context, where many students struggle with spoken English due to a lack of opportunities for practice. Finally, the study highlights the need for EFL teachers to be trained in the use of technology-enhanced language learning. While the e-tandem learners had positive attitudes toward the technology, some expressed reservations, and it is possible that these reservations may have affected their learning outcomes. EFL teachers in China may benefit from training in the use of technology-enhanced language learning, both to ensure that they can effectively integrate technology into their teaching and to address any concerns that students may have about the technology.

Despite the valuable insights obtained by this research, there might be some limitations which need to be taken into account while interpreting the outcomes. Firstly, the present sample size was relatively small, consisting of only postgraduate students in one university. As such, it is less likely to extend the findings to other EFL learners in various settings. Secondly, this research only concentrated on the effect of online language exchanges on speaking skills and WTC. Future researchers might explore the impact on other language skills, such as listening, reading, and writing, and other affective constructs that may influence language learning, such as motivation and anxiety. Additionally, the study did not consider the impact of cultural differences and their potential impact on the success of online language exchanges. Finally, future researchers can also explore the impact of various online language exchange programs and applications on language learning outcomes to identify the most effective tools and strategies for facilitating language learning through online exchanges.

## Data availability statement

The raw data supporting the conclusions of this article will be made available by the authors, without undue reservation. Requests to access these datasets should be directed to AZ, 20041029@zzut.edu.cn.

## Ethics statement

The studies involving human participants were reviewed and approved by School of Foreign Languages, Zhengzhou University of Technology, Zhengzhou, Henan. The patients/participants provided their written informed consent to participate in this study.

## Author contributions

AZ has completed the project and is the sole contributor.

## Conflict of interest

The author declares that the research was conducted in the absence of any commercial or financial relationships that could be construed as a potential conflict of interest.

## Publisher’s note

All claims expressed in this article are solely those of the authors and do not necessarily represent those of their affiliated organizations, or those of the publisher, the editors and the reviewers. Any product that may be evaluated in this article, or claim that may be made by its manufacturer, is not guaranteed or endorsed by the publisher.
